# Supplementary material for: Structural heterogeneity of the ion and lipid channel TMEM16F
Source: Nat Commun. 2024 Jan 2;15:110. doi: 10.1038/s41467-023-44377-7 (PMC10761740; doi:10.1038/s41467-023-44377-7)
Supplement: Supplementary file 1 — Supplementary Information [file 41467_2023_44377_MOESM1_ESM.pdf]

Supplementary Information

Structural heterogeneity of the ion and lipid channel TMEM16F

Zhongjie Ye[1,#], Nicola Galvanetto[2,#], Leonardo Puppulin[3,4], Simone Pifferi[1,5], Holger Flechsig[4], Melanie Arndt[2], Cesar Adolfo Sánchez Triviño[1], Michael Di Palma[5], Shifeng Guo[6,7], Horst Vogel[8,9], Anna Menini[1], Clemens Martin Franz[4], Vincent Torre[1,10,11*], Arin Marchesi[4,5*]

[1]International School for Advanced Studies (SISSA), 34136, Trieste, Italy.

[2]Deparment of Biochemistry and Department of Physics, University of Zurich, 8057 Zurich, Switzerland.

[3]Department of Molecular Sciences and Nanosystems, Ca' Foscari University of Venice, I-30172 Mestre, Venice, Italy

[4]WPI Nano Life Science Institute, Kanazawa University, Kakuma-machi, 920-1192, Kanazawa, Japan.

[5]Department of Experimental and Clinical Medicine, Università Politecnica delle Marche, 60126, Ancona, Italy.

[6]Shenzhen Key Laboratory of Smart Sensing and Intelligent Systems, Shenzhen Institute of Advanced Technology, Chinese Academy of Sciences, Shenzhen 518055, China

[7]Guangdong Provincial Key Lab of Robotics and Intelligent System, Shenzhen Institute of Advanced Technology, Chinese Academy of Sciences, Shenzhen 518055, China

[8]Shenzhen Institute of Advanced Technology, Chinese Academy of Sciences, 518055, Shenzhen, China

[9]Institut des Sciences et Ingénierie Chimiques (ISIC), Ecole Polytechnique Fédérale de Lausanne (EPFL), Lausanne, Switzerland

[10]Institute of Materials (ION-CNR), Area Science Park, Basovizza, 34149, Trieste, Italy.

[11]BIoValley Investments System and Solutions (BISS), 34148, Trieste, Italy.

[#] These authors contributed equally.

*Corresponding authors

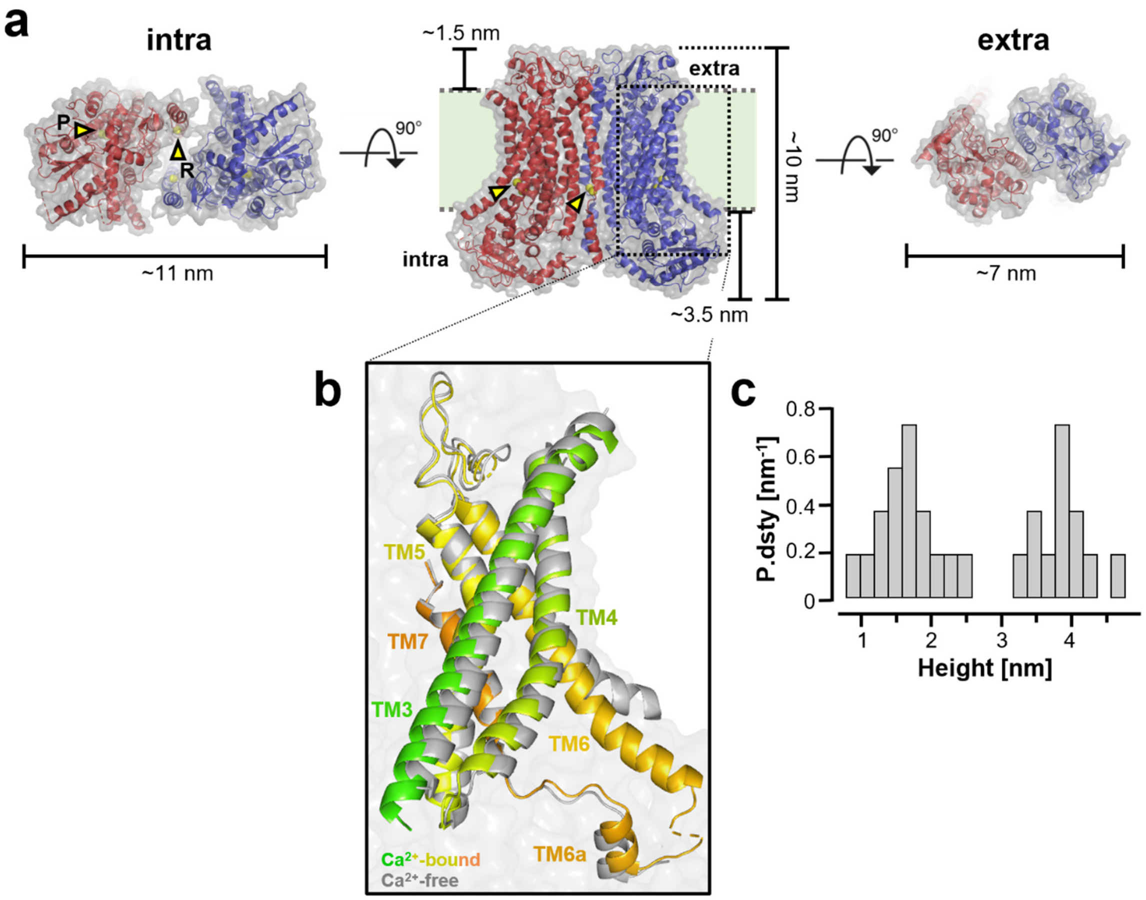

Supplementary Figure S1. The cryo-EM structures of TMEM16F. (a) High-resolution cryo-EM structure of the TMEM16F Ca^{2+}-bound state in digitonin (PDB 6QP6) viewed from the intracellular/cytosolic (left), membrane plane (middle), and extracellular (right) side. Ca^{2+} ions are shown as yellow spheres whereas arrowheads highlight the primary (P) and regulatory (R) binding sites. The molecular surface is shown in gray transparency while the two subunits composing the dimer are displayed as ribbon and colored in red and blue, respectively. **(b)** Inset showing a close-up view of the subunit cavity (TM3-TM7). Superposition of the Ca^{2+}-bound (PDB 6QP6, rainbow colors) and Ca^{2+}-free (PDB 6QPB, gray) structures reveal a tightly closed permeation pathway in either case, wherein ligand-induced transitions are limited to the intracellular half of TM6 and minor rearrangements of TM3 and TM4. Molecular models were rendered and aligned with PyMOL 2.5.4 (Schrödinger). **(c)** Height distribution of reconstituted proteins (n=28 molecules). The histogram exhibits a bimodal distribution with peaks of ~ 1.6nm and 3.8±0.4nm, well in agreement with the expected protrusion of ~1.5 and ~3.5nm for the extracellular and intracellular faces, respectively.

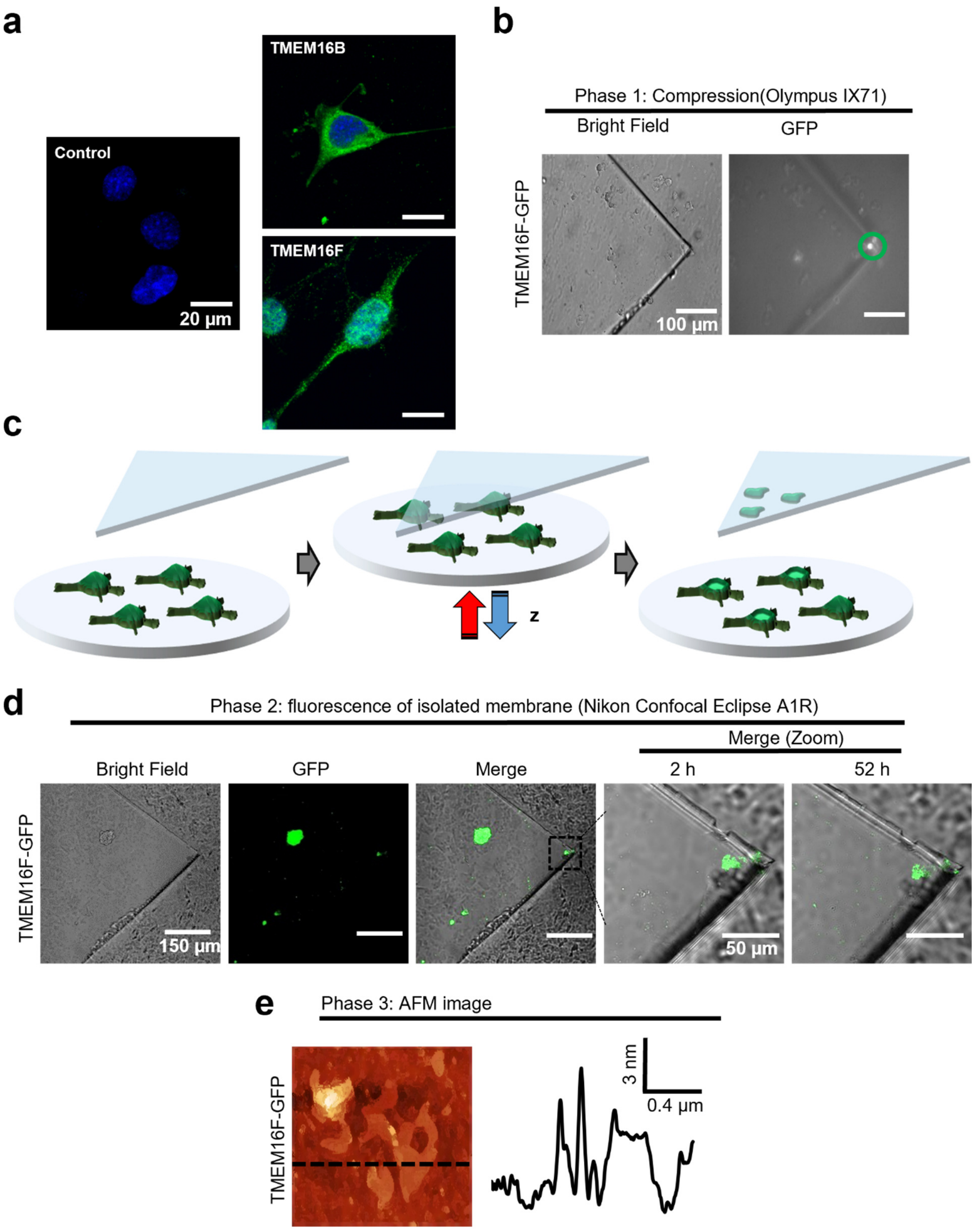

Supplementary Figure S2. Expression of TMEM16 proteins and isolation of native membranes. (a) Immunostaining of neuroblastoma NG108-15 cells treated with Abberior STARGREEN secondary antibody in the absence of a primary antibody (control, left panel), primary antibodies against TMEM16B (right-upper panel), and TMEM16F (right-bottom panel), demonstrating that both membrane proteins are abundantly expressed. In all the panels DAPI was used as nuclear marker. **(b,c)** Membranes were isolated by sandwiching

NG108-15 cells between the coverslip quarter coated with poly-lysine and the cell culture using the AFM stage stepper motor according to a previously described unroofing protocol[1–3]. Briefly, cells were brought into contact with the coverslip quarter by moving downward the AFM head, under close optical monitoring (**c**, left panel). Once in contact, the cell was left squeezed for ~0.5 min so that the apical membrane absorbed onto the coverslip quarter (**c**, mid panel). Afterwards, the AFM head was quickly lifted to achieve unroofing (**c**, right panel). The cultured cells shown in (**b**) were transfected with TMEM16F gene conjugated with GFP, whereas the targeted cell to be unroofed was marked by a green circle. The bright-field and the fluorescence images shown in **b** were captured by the inverted microscope-AFM system previously described[1]. **(d)** The presence of membrane fragments housing the GFP conjugated TMEM16s on the quarter were confirmed by confocal imaging up to 52 hours after the unroofing procedure (right-most panel). **(e)** A representative AFM image of isolated NG108-15 membrane patches (left) and the corresponding cross-sectional profile along the black dashed line (right).

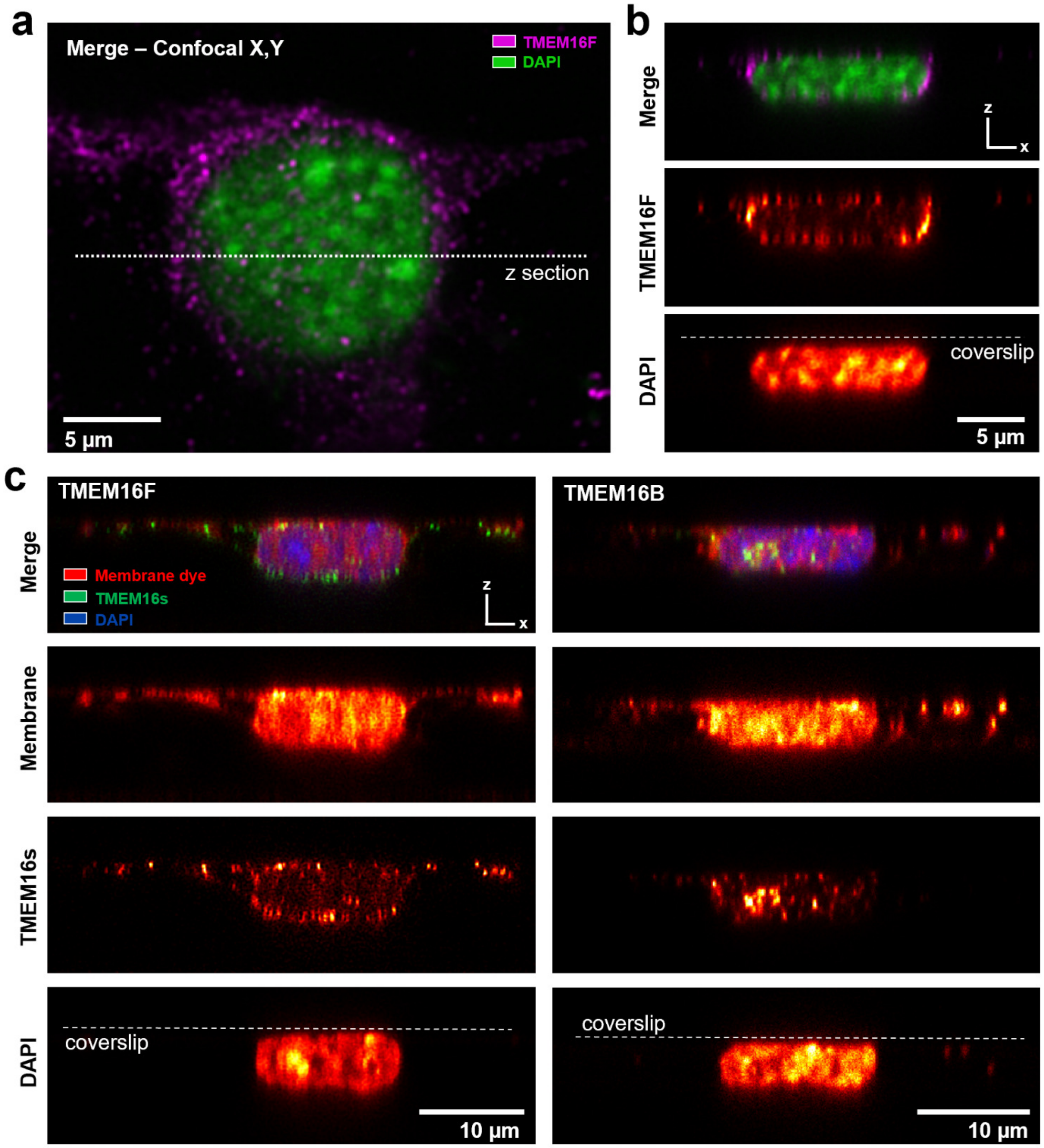

Supplementary Figure S3. Confocal analysis of the subcellular distribution of TMEM16F and TMEM16B. (a) XY confocal scan of neuroblastoma NG108-15 cells labelled in immunofluorescence with Abberior STARRED for TMEM16F and DAPI reveals dot-like distribution of TMEM16F at the plasma membrane. **(b)** XZ sections of TMEM16F expressing cells labelled with Abberior STARRED and DAPI in immunofluorescence reveal exclusion of TMEM16F from cytosol and low signal in the nucleus. **(c)** XZ sections of NG108-15 cells immunolabeled with primary antibodies against TMEM16F (left column) and TMEM16B (right column), membrane (Abberior Membrane STARRED), and nucleus (DAPI). TMEM16s labelling was developed with Abberior STARGREEN secondary antibody. Micrographs reveal colocalization of both proteins with the plasma membrane and confirm their exclusion from the cytosol. Occasional nuclear localization of TMEM16F or 16B was observed.

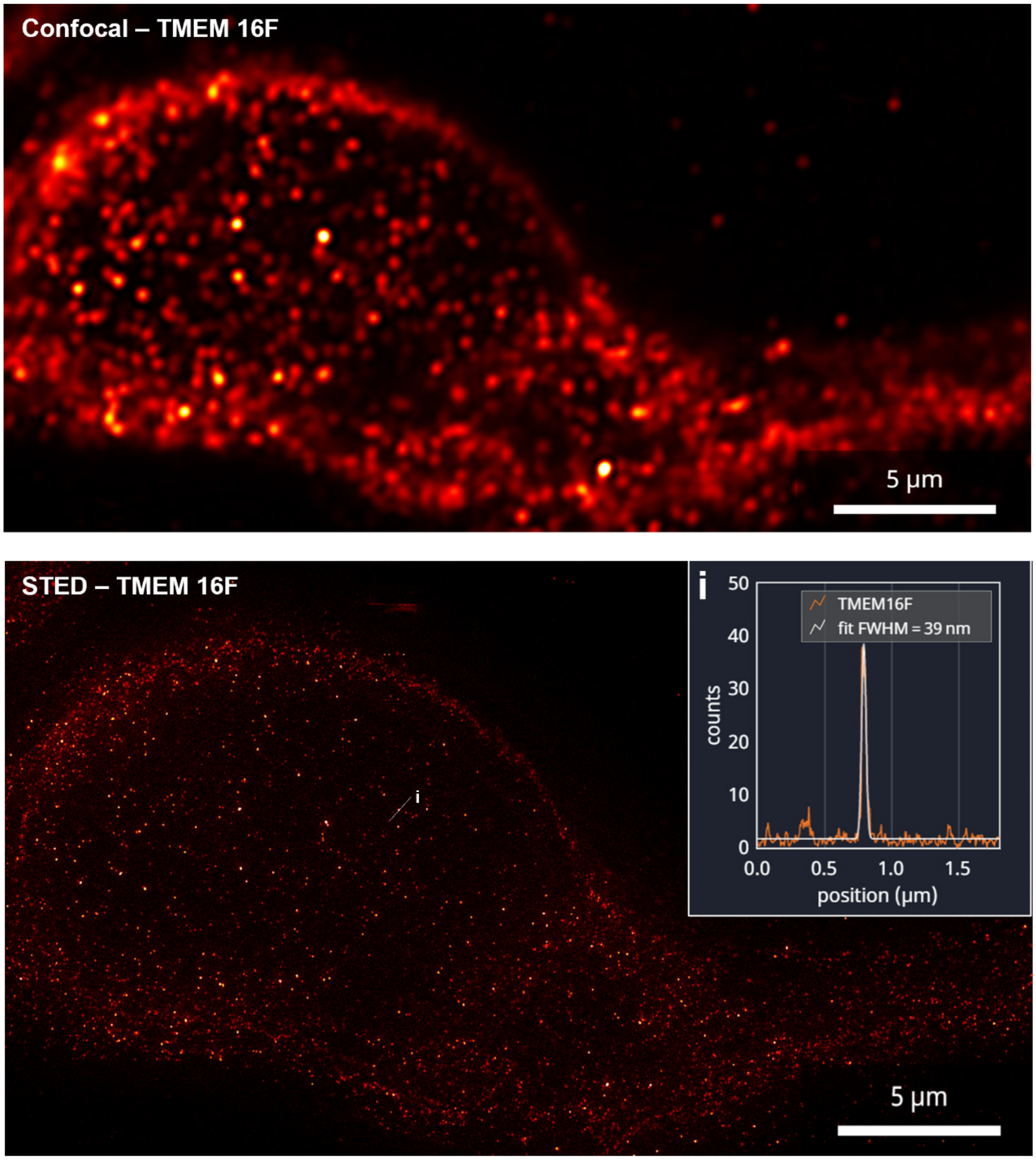

Supplementary Figure S4. Observation of TMEM 16F in stimulated emission depletion (STED) nanoscopy. Top: confocal observation of TMEM16F immunolabeled with Abberior STARRED at the plasma membrane of NG108-15 cells reveal a dense, yet spot-like distribution of the protein at the cell surface. Bottom: STED nanoscopy at resolution <40nm confirms that TMEM16F is not aggregated at the plasma membrane, nor generates a continuous signal within the cell. Inset: Gaussian fit on one typical spot of TMEM16F reveals a size within the resolution limit of the used STED microscope, which could be consistent with single TMEM16F molecules in complex with primary and secondary antibodies (the largest dimension of an antibody being 15nm).

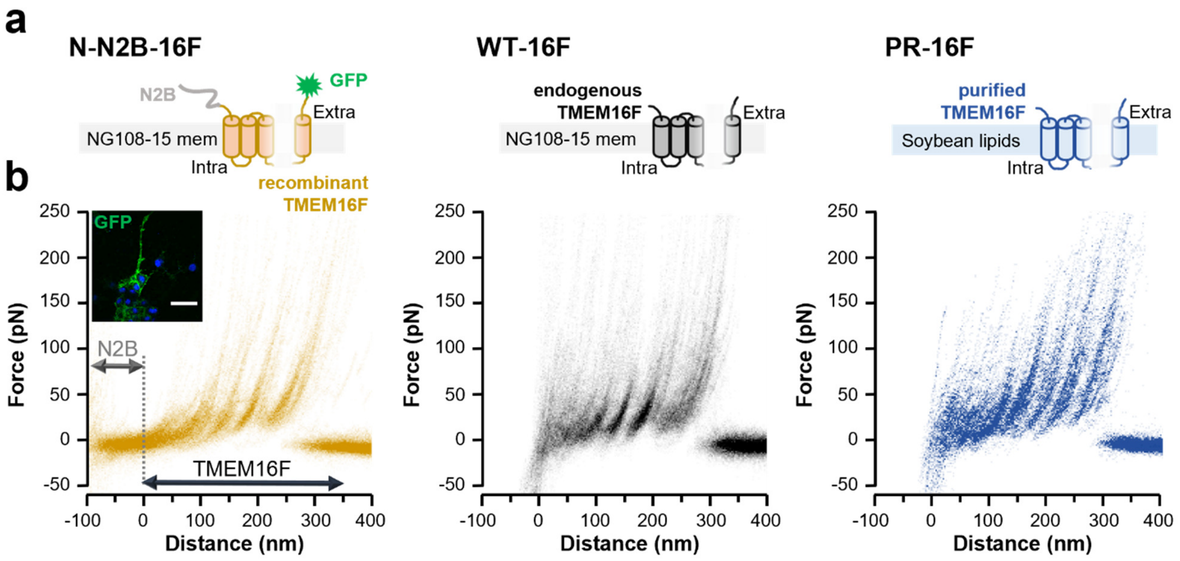

Supplementary Figure S5. Recombinant (N-N2B-16F), native (WT-16F) and reconstituted TMEM16F (PR-16F) show a similar unfolding pattern (a) Sketches of the three constructs used in the SMFS experiments. **(b)** Density plots of n=78, n=101 and n=68 FD curves (from left to right) of the constructs shown in **a**. TMEM16F proteins were unfolded in the absence of Ca^{2+}. The distinct N2B signature (double-headed arrow, left panel) is visible as an initial flat region of ~85 nm and identifies N-N2B-TMEM16F unfolded from the N-terminal end. Bayesian identification procedures were applied to find the native cluster of TMEM16F (WT-16F) that matched the unfolding pattern of N-N2B-TMEM16F (see Methods). In proteoliposomes housing reconstituted TMEM16F, we identified only one major unfolding pattern corresponding to WT-16F. No sawtooth-like spectra were observed in supported bilayers obtained from empty liposomes devoid of TMEM16F. The inset in **b** (left panel) shows a confocal image of NG108-15 cells overexpressing the N-N2B-16F construct. Hoechst labeled nuclei (blue) and the GFP signal (green) indicate TMEM16F membrane overexpression.

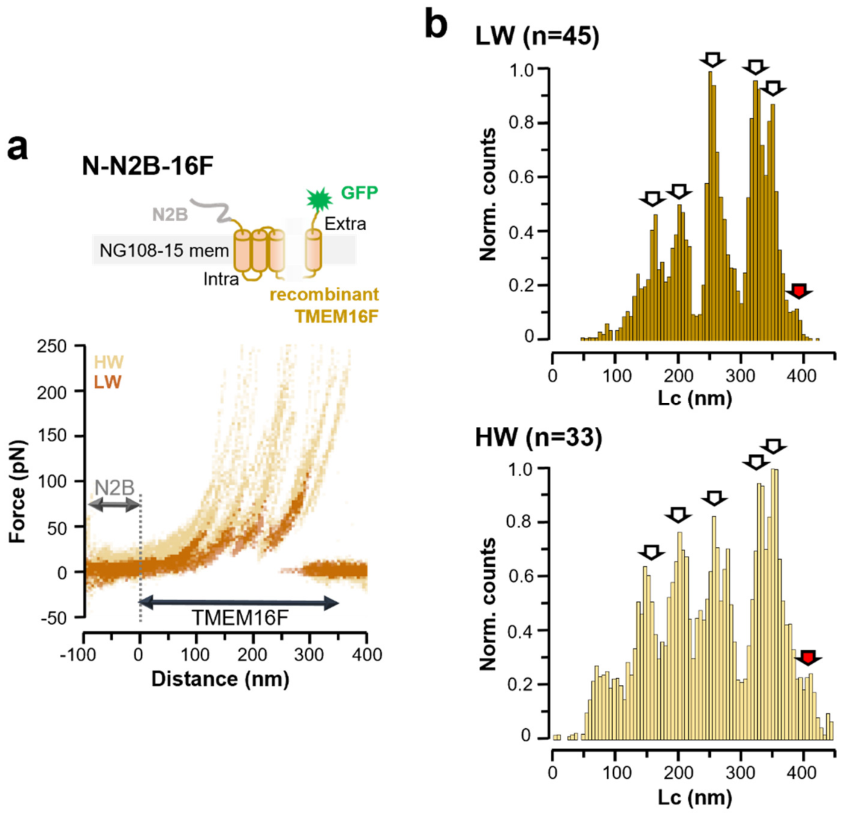

Supplementary Figure S6. TMEM16F unfolding with low and high work have similar contour lengths. **(a)** Superposition of representative F-D traces color coded according to their unfolding work in low (LW, dark ochre, n=13) and high work (HW, pale ochre, n=12). **(b)** Normalized contour length (Lc) histograms obtained after partitioning the F-D curves into LW (upper panel) and HW (lower panel) unfolding work. The force peaks have comparable positions (empty arrowheads) in both conditions, indicating an overall similar transmembrane topology and 3D architecture within the subunits. Some traces feature an additional force peak (red arrowhead, see Main text for details). The N2B-tagged TMEM16F were unfolded in the absence of Ca^{2+}.

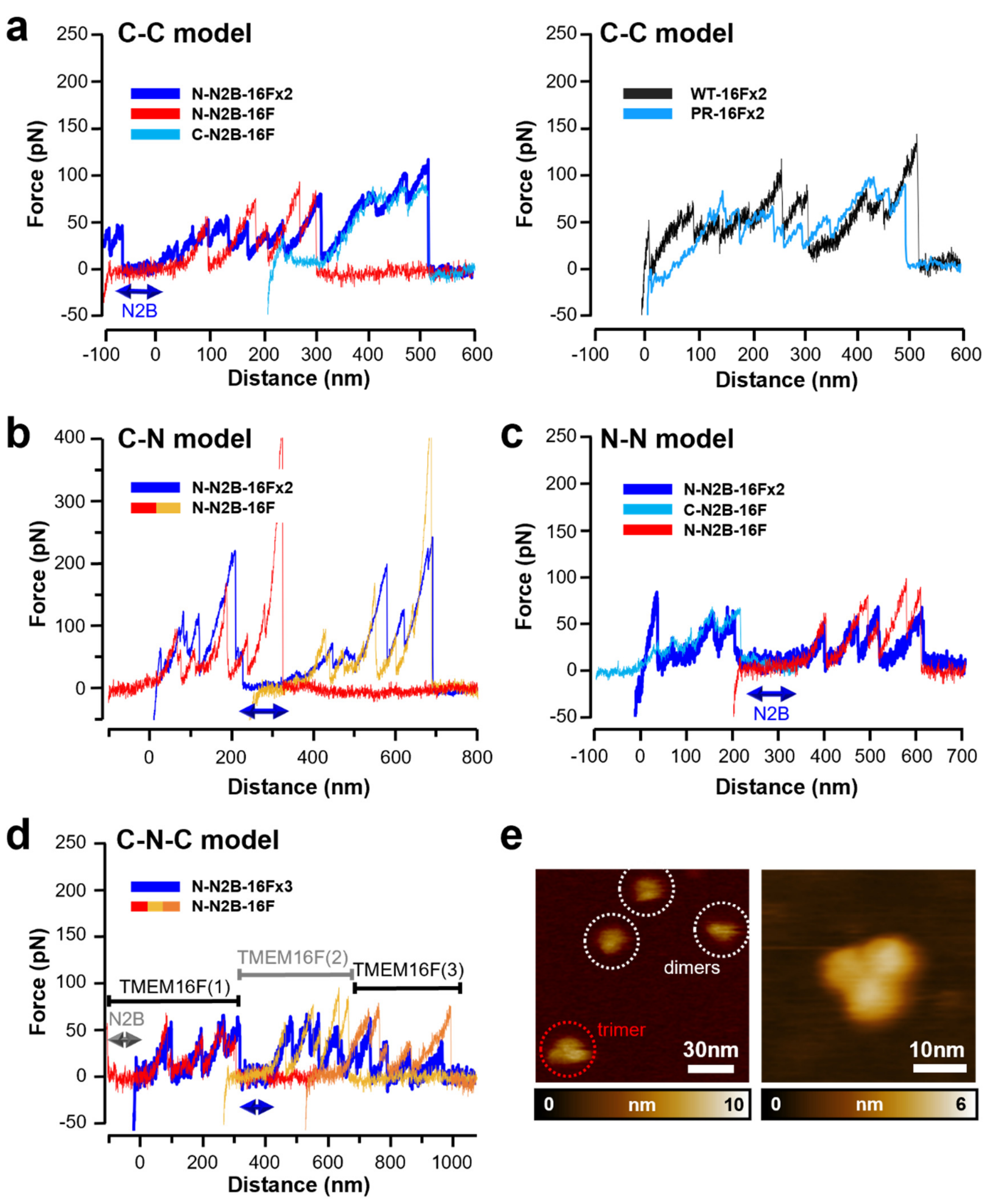

Supplementary Figure S7. TMEM16F displays different dimerization interfaces and oligomerization states. (a-c) Recombinant TMEM16F (N-N2B-16F) occasionally unfolded in tandem (N-N2B-16Fx2). The N2B characteristic ~85 nm long and flat unfolding segment (blue double-headed arrows) was observed both at the beginning (**a**) or midway through (**b,c**) the force-extension curve, indicating that TMEM16F protomers interact *via* their C-N (**b**) or N-N terminal domains (**c**). This hypothesis was further substantiated by aligning and adjoining to TMEM16F dimers representative monomer spectra wherein the N2B tag was conjugated either to the N- (N-N2B-16F in red/ochre colors) or C-terminus of TMEM16F (C-N2B-16F in cyan color). The N2B fingerprint aids identification of proteins attachment site to the AFM probe and assigns unfolding polarity (see main text for details). Unfolding of TMEM16F in tandem was also observed in SMFS experiments carried out on purified TMEM16F reconstituted in artificial membranes (**a**, right panel). WT-16Fx2 and PR-16Fx2

denote unfolding of TMEM16F dimers from non-transfected NG108-15 cell membranes and reconstituted TMEM16F, respectively. **(d)** Unfolding of putative trimeric TMEM16F assemblies (N-N2B-16Fx3) and alignment with representative N-N2B-16F monomers (red and light/dark ochre) indicating N- and C-terminal domain couplings. **(e)** HS-AFM overview (left panel) and high-resolution images (right panel) of reconstituted TMEM16F proteins confirmed the existence of trimeric assemblies alongside TMEM16F dimers. Unfolding (n=3) and imaging (n=2) of trimers were infrequent.

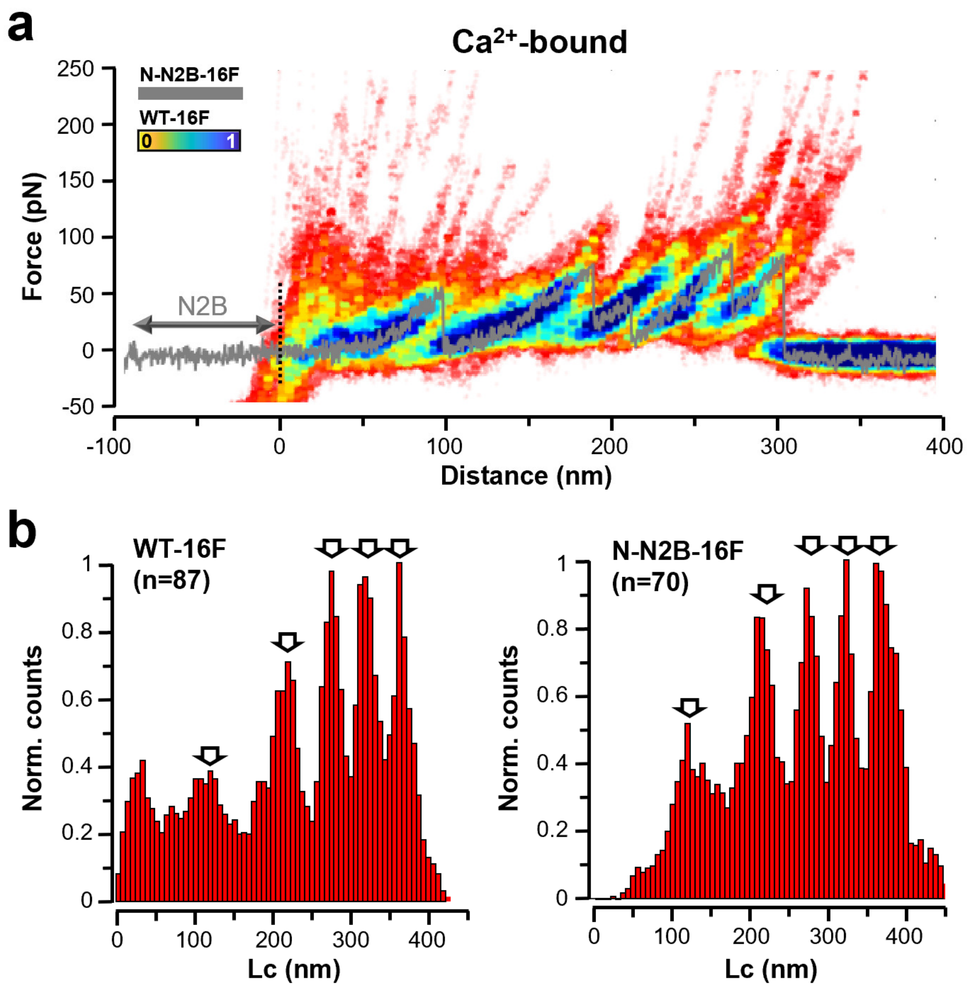

Supplementary Figure S8. Native (WT-16F) and recombinant TMEM16F (N-N2B-16F) have a similar unfolding pattern and force peak positions (a) Density plot of 87 F-D curves from WT-16F recorded in the presence of saturating Ca^{2+} (2 mM). One representative unfolding trace from Ca^{2+}-bound N-N2B-16F (gray line) is aligned and superimposed, indicating similar unfolding features. The double-headed arrow designates the unfolding of the N2B segment. **(b)** Normalized contour length (Lc) histograms obtained from 87 WT-16F (left panel) and 70 N-N2B-16F F-D curves (right panel) in the presence of Ca^{2+}. Force peaks associated to the unfolding of the transmembrane region (*i.e.* for Lc > 100 nm) have comparable contour length positions (empty arrowheads) in both proteins, indicating that the N-terminal attachment of N2B and the C-terminal GFP polypeptides did not affect TMEM16F mechanical properties.

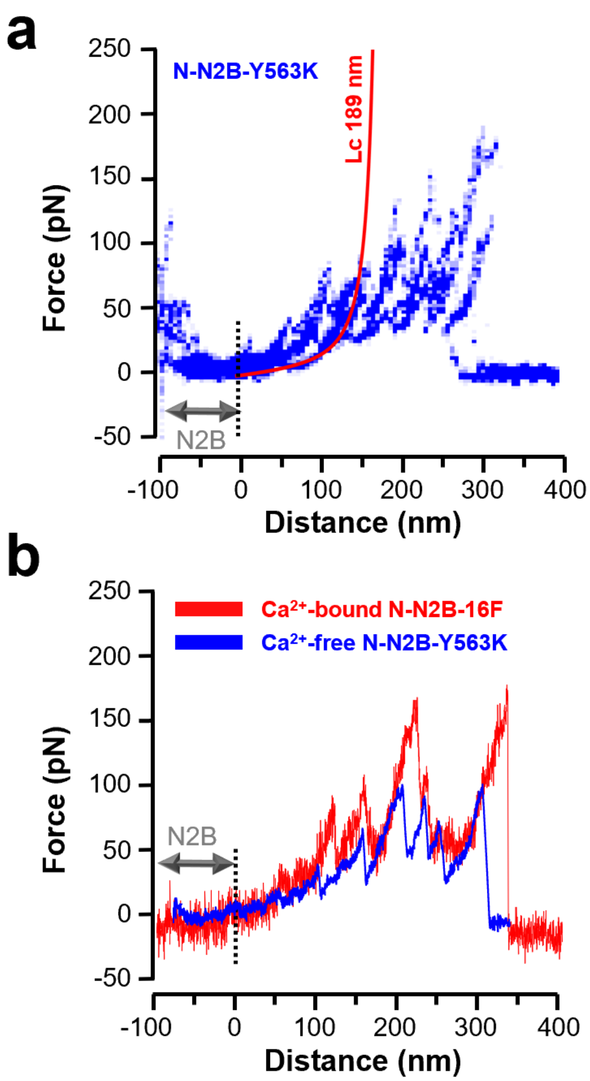

Supplementary Figure S9. Unfolding properties of the constitutively active Y563K mutant. (a) Density plot of six F-D traces from the constitutively scrambling mutant His_6-N2B-TMEM16F-Y563K-GFP (N-N2B-Y563K). The force peak corresponding to a contour length of 189 nm (red WLC fit) was observed in 6 out of 8 collected traces. **(b)** Superimposition of N-N2B-16F (red) and N-N2B-Y563K (blue) unfolding curves obtained in Ca^{2+}-bound and Ca^{2+}-free buffer, respectively. The similar position of force peaks suggests that N-N2B-16F was captured in the active state.

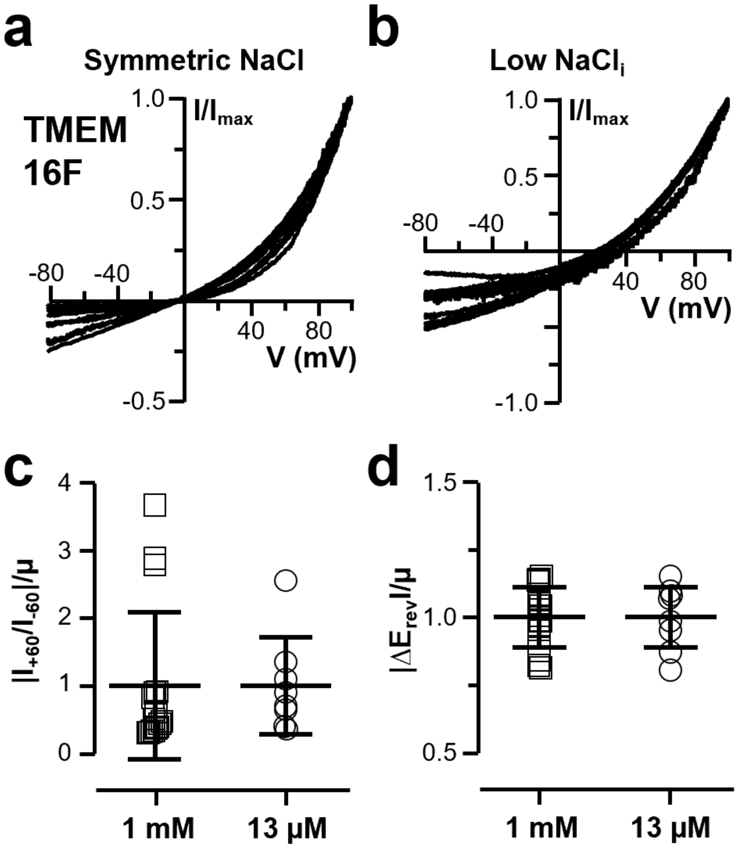

Supplementary Figure S10. The variability of electrical properties in TMEM16F channels. (a-b) The inside-out excised membrane patches expressing TMEM16F were recorded in symmetric NaCl (140 mM, **a**) and lower intracellular NaCl (14 mM, **b**) solutions. Their IV relations were determined via voltage ramps from -80 mV to +100 mV. Currents were activated by 13 µM $CaCl_2$. **(c)** Comparison of the changes of rectification for TMEM16F activated by the indicated Ca^{2+} concentrations. Rectification was calculated as the ratio between currents measured at +60 and -60 mV ($|I_{+60}/I_{-60}|$) and normalized to the average rectification value µ (n=16 for 1 mM, and n=8 for 13 µM). **(d)** Comparison of the shift of reversal potentials after the replacement of 140 mM NaCl with 14 mM NaCl normalized to the average value for TMEM16F activated by the indicated Ca^{2+} concentrations (n=19 for 1 mM and n=8 for 13 µM).

a

b

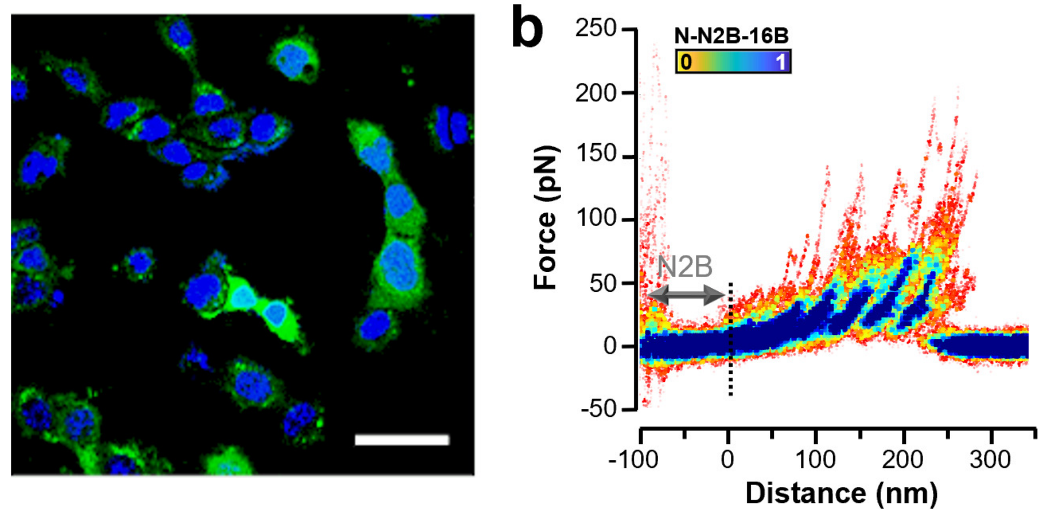

Supplementary Figure S11. TMEM16B overexpression and unfolding in the presence of saturating Ca^{2+}. (a) Overexpression of His_6-N2B-TMEM16B-GFP (N-N2B-16B) in NG108-15 cells**.** Hoechst labeled cell nuclei (blue) and the GFP emission signal (green) show that TMEM16B is mainly overexpressed on cell membrane. Scale bar 50 µm. **(b)** Density plot of 38 F-D curves from recombinant N-N2B-16B channels pulled in the presence of saturating Ca^{2+} (2 mM). The clear N2B signature (double-headed arrow) is visible as an initial flat region of ~85 nm and identifies TMEM16B channels unfolded from the N-terminal end.

```
TMEM16F     MQMMTRKVLLNMELEEDDDEDGDIVLE---NFDQTIVCPTFGSLENQQDFRTPEFEEFNG    57
TMEM16A     MRVPEK-------YSTLPAEDRSVHIVNICAIEDLGYLPSEGTLLNSLS-----VDPDAE    48
TMEM16B     ------------------------------------------------------------    0

                             Nβ1
TMEM16F     KPDSLFFTDGQRRIDFILVYEDESKKENNKK-----------------------------    88
TMEM16A     CKYGLYFRDGKRKVDYILVYHHKRASGSRTLARRGLQNDMVLGTRSVRQDQPLPGKGSPV    108
TMEM16B     ----MHFHDNQRKVDYVLAYHYRKRGAHLGHGSPGHSLAVISNG-ETGKER---HGGGPG    52
                :.* *.:*::*::*.*. .

                         Nα1                 Nβ2          Nβ3       Nα2
TMEM16F     ----------GTNEKQKRKRQAYESNLICHGLQLEATRSVSDDKLVFVKVHAPWEVLCTY    138
TMEM16A     DAGSPEVPMDYHEDDKRFRREEYEGNLLEAGLELENDEDTKIHGVGFVKIHAPWHVLCRE    168
TMEM16B     --DVELGPLDALEEERREQRDEFEHNLMAAGLELEKDLESKSQGSVFVRIHAPWQVLARE    110
                        ::.:: :*: :* **:  **:**   . . .   **::****.**.

TMEM16F     AEIMHIKLPLKPNDLKTRSPFGNLNWFTKVLRVNESVIKP--------EQEFFTAPFEKS    190
TMEM16A     AEFLKLKMPTKKVYHI-SETRGLLKTINSVLQKITDPIQPKVAEHRPQTTKRLSYPFSRE    227
TMEM16B     AEFLKIKVPTKKMYEI-KAGGSIAKKFSAILQTLSSPLQPRVPEHSNNRMKNLSYPFSRE    169
            **:::::*:* *            .  : :. :*:   . ::*          : :: **.:.

                          Nα3                     Nα4       Nβ4
TMEM16F     RMNDFYILDRDSFFNPATRSRIVYFILSRVKYQVMNNVNKFGINRLVSSGIYKAAFPLHD    250
TMEM16A     KQHLFDLTDRDSFFDSKTRSTIVYEILKRTTCTK--AKYSMGITSLLANGVYSAAYPLHD    285
TMEM16B     KMYLYNIQEKDTFFDNATRSRIVHEILKRTACSR--ANNTMGINSLIANNIYEAAYPLHD    227
            :   : : ::*:**:  *** **: **.*.           .:**. *::..:*.**:****

                         pre-TM1a           pre-TM1b      TM1
TMEM16F     CRFNYESEDISCPSERYLLYREWAHPRSIYKKQPLDLIRKYYGEKIGIYFAWLGYYTQML    310
TMEM16A     GDYEGDNVEF---NDRKLLYEEWASYGVFYKYQPIDLVRKYFGEKVGLYFAWLGAYTQML    342
TMEM16B     GEYDSPGDDM---NDRKLLYQEWARYGVFYKFQPIDLIRKYFGEKIGLYFAWLGLYTSFL    284
              ::  . ::   .:* ***.***    :** **:**:***:***:*:****** **.:*

                  TM1            TM1a                          TM1b
TMEM16F     LLAAVVGVACFLYGYLDQDNCTWSKEVCDPDIGGQILMCPQCDRLCPFWRLNITCESSKK    370
TMEM16A     IPASIVGVIVFLYGCATVDENIPSMEMCDQR--YNITMCPLCDKTCSYWKMSSACATARA    400
TMEM16B     IPSSVIGVIVFLYGCATIEEDIPSKEMCDHQ--NAFTMCPLCDKSCDYWNLSSACGTARA    342
            : ::::**  ****    ::    * *:**        : *** **: * :*.:. :* :::

                            TM2                                   TM2a
TMEM16F     LCIFDSFGTLIFAVFMGVWVTLFLEFWKRRQAELEYEWDTVELQQEE-----QARPEYEA    425
TMEM16A     SHLFDNPATVFFSVFMALWAATFMEHWKRKQMRLNYRWDLTGFEEED----HPRAEYEA    456
TMEM16B     SHLFDNPATVFFSIFMALWATMFLENWKRLQMRLGYFWDLTGIEEEEERSQEHSRPEYET    402
              :**. .*::*::**.:*.: *:* ***  * .* * ** . :::**      : * ***:

                                                         TM3
TMEM16F     QCNHVVINEITQ--------------EEERIPFTTCGKCIRVTLCASAVFFWILLIIASV    471
TMEM16A     RVLEKSLRKE------------SRNKETDKVKLTWRDRFPAYFTNLVSIIFMIAVTFAIV    504
TMEM16B     KVREKLLKESGKSAVQKLEANSPEDDEDDEDKLTWKDRFPGYLMNFASILFMIALTFSIV    462
            :  .  :.:                  * :.  :*  .:         :::* * : :: *

                  TM3                                  TM4
TMEM16F     IGIIVYRLSVFIVFSTTLPKNPNGTDPIQKYLTPQMATSITASIISFIIIMILNTIYEKV    531
TMEM16A     LGVIIYRISTAAALAMN--SSPSVRSN------IRVTVTATAVIINLVVILLDEVYGCI    556
TMEM16B     FGVIVYRITTAAALSLN--K--ATRSN------VRVTVTATAVIINLVVILILDEIYGAV    512
            :*:*:**::.  .:: .  .     .      ::::.: ** **.:::*::*: :*  :

                                   TM5
TMEM16F     AIMITNFELPRTQTDYENSLTMKMFLFQFVNYYSSCFYIAFFKGKFVGYPGDPVYLLGKY    591
TMEM16A     ARWLTKIEVPKTEKSFEERLTFKAFLLKFVNSYTPIFYVAFFKGRFVGRPGDYVYIFRSF    616
TMEM16B     AKWLTKIEVPKTEQTFEERLILKAFLLKFVNAYSPIFYVAFFKGRFVGRPGSYVYVFDGY    572
            *  :*::*:*:*:  :*: * :* **::*** *:  **:*****:*** **. **::  :
                                           563
```

15

```
                 TM6
TMEM16F    RSEECDPGGCLLELTTQLTIIMGGKAI-WNNIQEVLLPWVMNLIGRYKRVS-------GS   643
TMEM16A    RMEECAPGGCLMELCIQLSIIMLGKQLIQNNLFEIGIPKMKKFIRYLKLRRQSPSDREEY   676
TMEM16B    RMEECAPGGCLMELCIQLSIIMLGKQLIQNNIFEIGVPKLKKLFRKLKDETEPGESDPDH   632
           * *** *****:**  **:*** ** :  **: *: :* : :::    *

              TM6a            TM7                  TM8
TMEM16F    EKITPRWEQDYHLQPMGKLGLFYEYLEMIIQFGFVTLFVASFPLAPLLALVNNILEIRVD   703
TMEM16A    VKRKQRYEVDFNLEPFA--GLTPEYMEMIIQFGFVTLFVASFPLAPLFALLNNIIEIRLD   734
TMEM16B    SKRPEQWDLDHSLEPYT--GLTPEYMEMIIQFGFVTLFVASFPLAPVFALLNNVIEVRLD   690
            *   ::: *. *:*     **  **:*******************::**:**::*:*:*

                                  TM9                post-TM9α1
TMEM16F    AWKLTTQFRRMVPEKAQDIGAWQPIMQGIAILAVVTNAMIIAFTSDMIPRLVYYWSFSIP   763
TMEM16A    AKKFVTELRRPVAIRAKDIGIWYNILRGVGKLAVIINAFVISFTSDFIPRLVYLYMYSQN   794
TMEM16B    AKKFVTELRRPDAVRTKDIGIWFDILSGIGKFSVIINAFVIAVTSDFIPRLVYQYSYSHN   750
           * *:.*::**    :::*** *  *: *:. ::*: **::*:.***:****** : :*

                           α2   β1              α3    β2
TMEM16F    PYGDHTYYTMDGYINNTLSVFNITDFKNTDKENPYIGLG-NYTLCRYRDFRNPPGHPQEY   822
TMEM16A    -------GTMHGFVNHTLSSFNVSDFQNGTAPNDPLDLGYEVQICRYKDYREPPWSEHKY   847
TMEM16B    -------GTLHGFVNHTLSFFNVSQLKEGTQPENS-QFDQEVQFCRFKDYREPPWAPNPY   802
                  *:.*::*:*** **:::::    :    :. :  :**::*:*:**    : *

                    TM10                      Cα1
TMEM16F    KHNIYYWHVIAAKLAFIIVMEHIIYSVKFFISYAIPDVSKITKSKIKREKYLTQKLLHE-   881
TMEM16A    DISKDFWAVLAARLAFVIVFQNLVMFMSDFVDWVIPDIPKDISQQIHKEKVLMVELFMRE   907
TMEM16B    EFSKQYWSVLSARLAFVIIFQNLVMFLSVLVDWMIPDIPTDISDQIKKEKSLLVDFFLKE   862
           . .  :* *::*:***:*:::::  :. ::.: ***: .  ..:*::** *  .:: .

TMEM16F    SHLKDLTKNMGIIAERIGGTVDNSVRPKLE----------------------   911
TMEM16A    EQGKQQLLDTWMEKEKPRDVPCNNHSPTTHPEAGDGSP---VPSYEYHGDAL   956
TMEM16B    EHEKVKLADEPTQRSQGGGDRSRRSRAASSAPSGRSQPGSIASSGSQHTNV-   913
           .: *    :     .:   .    .
```

Supplementary Figure S12. Multiple sequences alignment of TMEM16A/B/F paralogues. Protein sequences of *Mus musculus* TMEM16F (Ano6, NP_780553.2), TMEM16A (Ano1, NP_001229278.2), and TMEM16B (Ano2, NP_705817.1) were aligned with Clustal Omega[4]. The transmembrane helices of TMEM16F (light green) and TMEM16A (red) are colored according to their cryo-EM structures[5,6]. The NCD is highlighted only for TMEM16F sequence (cyan and violet for α-helices and β-sheets, respectively). Secondary structure elements are indicated on top of the sequence alignment and refer to mTMEM16F. Dotted lines define disordered segments in the cryo-EM structure (PDB 6QPB). The position of TMEM16F residue 563 which Y/K substitution results in a gain-of-function mutant is also underlined.

Secondary structure		Polypeptide segment	Length in a.a.	Average contour length (Lc) in nm		Probability	
				Ca^{2+}-free	Ca^{2+}-bound	Ca^{2+}-free	Ca^{2+}-bound
Cytoplasmic N-terminus	Nβ1	F73-E78	6				
	Nα1	T90-H108	19				
	Nβ2	Q111-R116	6				
	Nβ3	L123-H129	7				
	Nα2	W132-H143	12				
	Nα3	P206-R219	14				
	Nα4	I232-S238	7				
	Nβ4	A244-F246	3				
Pre-TM1	a	E265-E272	8				
	b	L285-Y292	6				
Helix TM1		E294-D327	34				
Loop TM1-TM2		Q328-S376	49				
Helix TM2		F377-W408	32	161±15	156±14	0.68	0.46
Loop TM2-TM3		D409-T445	37				
Helix TM3		T446-T487	42		189±5		0.33
Loop TM3-TM4		T488-T504	17				
Helix TM4		P505-F538	34				
Loop TM4-TM5		E539-T543	5	212±11		0.97	
Helix TM5		Q544-F572	29				
Loop TM5-TM6		F573-C601	29		227±13		0.81
Helix TM6		L602-Y637	36				
Loop TM6-TM7		K638-L664	27	259±11		0.97	
Helix TM7		F665-L680	16				
Loop TM7-TM8		F681-L687	7		276±11		0.97
Helix TM8		A688-T709	22				
Loop TM8-TM9		Q710-A724	15	288±5		0.40	
Helix TM9		W725-F746	22				
Loop TM9-TM10		T747-N824	78	323±12	327±10	0.69	0.94
Helix TM10		I825-A856	32				
Loop TM10-Cα1		I857-S861	5				
Cα1		K862-L874	13				
Cytosolic C-terminus		L875-E911	37	360±14 (Dtc)	377±13 (Dtc)	-	-

Supplementary Table 1. Allocation of the unfolding peaks and associated probabilities to the primary sequence and secondary structure elements of TMEM16F channels in the active (Ca^{2+}-bound) and resting (Ca^{2+}-free) states. Structural elements were annotated according to the TMEM16F cryo-EM structure (PDB 6QPB). Lc values and probabilities were obtained from fitting normalized Lc histograms shown in Fig. **4a,c** with multiple gaussian distributions (see Methods section for details).

Plasmids	Vector	Provider/Company
peGFP-N1		Prof. Guidalberto Manfioletti
mTMEM16A-GFP	peGFP-N1	Prof. Criss Hartzell
mTMEM16B	Sport6	RZPD
mTMEM16F-GFP	peGFP-N1	Prof. Lily Jan
mTMEM16F-Y563K-GFP	peGFP-N1	Prof. Huanghe Yang
His_6-N2B-mTMEM16B-GFP	peGFP-N1	GENEWIZ
His_6-N2B-mTMEM16F-GFP	peGFP-N1	GENEWIZ
His_6-N2B-mTMEM16F-Y563K-GFP	peGFP-N1	GENEWIZ
GFP-mTMEM16F-N2B-His_6	peGFP-C1	GENEWIZ

Supplementary Table 2. List of plasmids. Constructs conjugated with His_6-N2B/N2B-His_6 fingerprint and GFP tag were prepared by Genewiz, Suzhou, China. All plasmids were confirmed by sequencing and/or restriction enzyme analysis.

Supplementary References

1. Galvanetto, N. Single-cell unroofing: probing topology and nanomechanics of native membranes. *Biochim Biophys Acta Biomembr* **1860**, 2532–2538 (2018).
2. Galvanetto, N. *et al.* Unfolding and identification of membrane proteins in situ. *Elife* **11**, e77427 (2022).
3. Xu, J., Galvanetto, N., Nie, J., Yang, Y. & Torre, V. Rac1 Promotes Cell Motility by Controlling Cell Mechanics in Human Glioblastoma. *Cancers* **12**, 1667 (2020).
4. Sievers, F. *et al.* Fast, scalable generation of high-quality protein multiple sequence alignments using Clustal Omega. *Mol Syst Biol* **7**, 539 (2011).
5. Alvadia, C. *et al.* Cryo-EM structures and functional characterization of the murine lipid scramblase TMEM16F. *Elife* **8**, e44365 (2019).
6. Dang, S. *et al.* Cryo-EM structures of the TMEM16A calcium-activated chloride channel. *Nature* **552**, 426–429 (2017).
