# Supplementary material for: Structural heterogeneity of the ion and lipid channel TMEM16F
Source: Nat Commun. 2024 Jan 2;15:110. doi: 10.1038/s41467-023-44377-7 (PMC10761740; doi:10.1038/s41467-023-44377-7)
Supplement: Supplementary file 3 — Description of Additional Supplementary Files [file 41467_2023_44377_MOESM3_ESM.pdf]

Supplementary Movie 1. HS-AFM movie of mTMEM16F subunit dynamics. Imaging was performed at room temperature (24-26°C) in liquid (150 mM NaCl, 20 mM HEPES, 2 mM $CaCl_2$, pH7.4) at 4 frame/s. Left: Raw movie. Right: Filtered movie with a Gaussian 2D-space operator (standard deviation: 0.75 pixels; kernel size: 3x3 pixels). Pixel sampling: 0.4 nm per pixel. Full image depth: 6 nm. Scale bar: 10 nm. Movie played back at 30 frames/s.

Supplementary Movie 2. HS-AFM movie of Ca^{2+}-induced mTMEM16F transitions and lipid bilayer remodelling. Imaging was performed at room temperature (24-26°C) in liquid (150 mM NaCl, 20 mM HEPES, 1 mM EDTA, pH7.4) at 3.64 (frames #1-300) and 5 frame/s (frames #301-2000). $CaCl_2$ was added at a final effective concentration of ~2 mM, as indicated. Left: Contrast-adjusted movie to a full-height scale of 6 nm. Right: Contrast-adjusted movie to a full-height scale of 2 nm. Pixel sampling: 0.375 nm per pixel. Scale bar: 10 nm. Movie played back at 30 frames/s.
