# Supplementary material for: Structural heterogeneity of the ion and lipid channel TMEM16F
Source: Nat Commun. 2024 Jan 2;15:110. doi: 10.1038/s41467-023-44377-7 (PMC10761740; doi:10.1038/s41467-023-44377-7)
Supplement: Supplementary file 6 — Reporting Summary [file 41467_2023_44377_MOESM6_ESM.pdf]

nature portfolio

Corresponding author(s): Arin Marchesi
Vincent Torre

Last updated by author(s): Nov 30, 2023

Reporting Summary

Nature Portfolio wishes to improve the reproducibility of the work that we publish. This form provides structure for consistency and transparency in reporting. For further information on Nature Portfolio policies, see our Editorial Policies and the Editorial Policy Checklist.

Statistics

For all statistical analyses, confirm that the following items are present in the figure legend, table legend, main text, or Methods section.

n/a	Confirmed	
☐	☒	The exact sample size (*n*) for each experimental group/condition, given as a discrete number and unit of measurement
☐	☒	A statement on whether measurements were taken from distinct samples or whether the same sample was measured repeatedly
☒	☐	The statistical test(s) used AND whether they are one- or two-sided<br>*Only common tests should be described solely by name; describe more complex techniques in the Methods section.*
☒	☐	A description of all covariates tested
☒	☐	A description of any assumptions or corrections, such as tests of normality and adjustment for multiple comparisons
☐	☒	A full description of the statistical parameters including central tendency (e.g. means) or other basic estimates (e.g. regression coefficient) AND variation (e.g. standard deviation) or associated estimates of uncertainty (e.g. confidence intervals)
☒	☐	For null hypothesis testing, the test statistic (e.g. *F*, *t*, *r*) with confidence intervals, effect sizes, degrees of freedom and *P* value noted<br>*Give P values as exact values whenever suitable.*
☒	☐	For Bayesian analysis, information on the choice of priors and Markov chain Monte Carlo settings
☒	☐	For hierarchical and complex designs, identification of the appropriate level for tests and full reporting of outcomes
☒	☐	Estimates of effect sizes (e.g. Cohen's *d*, Pearson's *r*), indicating how they were calculated

Our web collection on statistics for biologists contains articles on many of the points above.

Software and code

Policy information about availability of computer code

Data collection	1. JPKSPM (JPK/Brucker) acquisition software for SMFS-AFM data collection;<br>2. Laboratory-developed software based on Igor Pro 8 (WaveMetrics) and Visual Basic.NET (Microsoft) for HS-AFM data collection<br>3. Clampex vers.9 (pCLMAP 9, Molecular Devices) for electrophysiology data collection<br>4. NIS-Elements (Nikon) and STEDYCON (Abberior GmbH) acquisition software for confocal and STED imaging

Data analysis	1. Matlab R2017b and R2022a (MathWorks);<br>2. ImageJ 1.47v and 1.52e (NIH);<br>3. PyMOL vers. 2.5.4 (Schrodinger);<br>5. Gwyddion vers. 2.58 (64bit).<br>6. Published code developed in Matlab and available in public repositories or from the authors:<br>-https://github.com/arin83/U1067/;<br>-https://github.com/galvanetto/Fodis (Fodis software);<br>-https://github.com/ninailieva/SMFS_clustering;<br>7. IgorPro vers 6.3 and vers. 8 (WaveMetrics)<br>8. Microsoft Excel 365<br>9. BioAFMviewer (https://www.bioafmviewer.com/)<br>10. Image Gallery vers. 9 (Abberior GmbH)<br>11. ImageJ plugin Template Matching and Slice Alignment (64 bit)

For manuscripts utilizing custom algorithms or software that are central to the research but not yet described in published literature, software must be made available to editors and reviewers. We strongly encourage code deposition in a community repository (e.g. GitHub). See the Nature Portfolio guidelines for submitting code & software for further information.

Data

Policy information about availability of data

All manuscripts must include a data availability statement. This statement should provide the following information, where applicable:

- Accession codes, unique identifiers, or web links for publicly available datasets
- A description of any restrictions on data availability
- For clinical datasets or third party data, please ensure that the statement adheres to our policy

The manuscript figures, supplementary information, and source data files contain all data necessary to interpret, verify, and extend the presented work. The raw AFM data is saved as .jpk and .asd files and therefore can only be opened using proprietary software. These data are available from n.galvanetto@bioc.uzh.ch (SMFS dataset) and a.marchesi@staff.univpm.it (HS-AFM data) upon request. The source data underlying Figures 1c,e-i, 2b,e, 3a-c, 4a-f, 5a-h, and Supplementary Figures S1c, S2e, S6b, S7a-d, S8b, S9b, S10a-d are provided as a Source Data file. The following published PDB codes were used: 6P46 [https://doi.org/10.2210/pdb6P46/pdb], 6QP6 [https://doi.org/10.2210/pdb6QP6/pdb], 6QPB [https://doi.org/10.2210/pdb6QPB/pdb]

Research involving human participants, their data, or biological material

Policy information about studies with human participants or human data. See also policy information about sex, gender (identity/presentation), and sexual orientation and race, ethnicity and racism.

Reporting on sex and gender	N/A
Reporting on race, ethnicity, or other socially relevant groupings	N/A
Population characteristics	N/A
Recruitment	N/A
Ethics oversight	N/A

Note that full information on the approval of the study protocol must also be provided in the manuscript.

Field-specific reporting

Please select the one below that is the best fit for your research. If you are not sure, read the appropriate sections before making your selection.

☒ Life sciences ☐ Behavioural & social sciences ☐ Ecological, evolutionary & environmental sciences

For a reference copy of the document with all sections, see nature.com/documents/nr-reporting-summary-flat.pdf

Life sciences study design

All studies must disclose on these points even when the disclosure is negative.

Sample size	No statistical methods were used to determine semple size in HS-AFM, electrophysiology and immunofluorescence experiments. The minimum sample size in SMFS-AFM experiments is based on the criterion that the standard error of the mean of the position of the unfolding peaks in a cluster is at least ten times smaller than their separation (which is usually in the order of 20–40 nm). The standard deviation of these peaks is usually about 10 nm, therefore the minimum sample size according to our criterion is ~25 FD curves.

Data exclusions	Leaky recordings (seal resistance less than 1 GigaOhm) were discarded (electrophysisiology). No othere exclusion criteria was used
Replication	SMFS-AFM analyses performed on randomized subsets of the collected data generate the same results (same clusters, proportionally less populated).<br>HS-AFM experiments were independently replicated (from 3 to 5 times depending on the experiments) on two different reconstitution batches, on different days, and with different AFM tips.<br>Electrophysiology experiments were performed on multiple cells/patches (from 6 to 19 depending on the experiments) from at least two different transfections. All attempts at replication were successful, although results for TMEM16F showed large variability from experiment to experiment (as shown in Fig. 5a,b and discussed in the manuscript).<br>Immunofluorescence and fluorescence imaging (epifluorescence, confocal, and STED) were performed in triplicate and all replication attempts were succesfull.
Randomization	Not relevant. The study did not allocate experimental groups.
Blinding	Blinding was not performed. No a priori knowledge could be assumed about the present observations, and blinding is therefore not applicable. Data were analyzed systematically through an objective analysis workflow, as described in the manuscript.

Reporting for specific materials, systems and methods

We require information from authors about some types of materials, experimental systems and methods used in many studies. Here, indicate whether each material, system or method listed is relevant to your study. If you are not sure if a list item applies to your research, read the appropriate section before selecting a response.

Materials & experimental systems

n/a	Involved in the study	
☐	☒	Antibodies
☐	☒	Eukaryotic cell lines
☒	☐	Palaeontology and archaeology
☒	☐	Animals and other organisms
☒	☐	Clinical data
☒	☐	Dual use research of concern
☒	☐	Plants

Methods

n/a	Involved in the study	
☒	☐	ChIP-seq
☒	☐	Flow cytometry
☒	☐	MRI-based neuroimaging

Antibodies

Antibodies used	1. 1:1000 for Hoechst（Cat# 33342, Thermo Scientific） and DAPI (Cat# 32670, Sigma);<br>2. 1:400 for TMEM16B（Cat# 20647-1-AP, Proteintech Euro, Polyclonal, Lot# 00013055） and TMEM16F (Cat# ACL-016, Alomone Labs, Polyclonal, Lot# AB_2756564)<br>3. 1:800 for Alexa 594-labeled goat anti-rabbit (Cat# A11037, Invitrogen, Polyclonal).<br>4. 1:200 Abberior STARRED membrane* (Cat# STRED-0206-100PMOL, Lot# 11124SN-1), STARRED* (Cat# STRED-1002-20UG, Lot# 20808PK-8) and STARGREEN* (Cat# STGREEN-1002-20UG, Lot# 21028PK-2) goat anti-rabbit<br>*kind gift from Abberior GmbH
Validation	All the antibodies (TMEM16B and TMEM16F) were bought from companies or commercial vendors, and were validated by the manufacturers by different assays:<br><br>Anti-TMEM16B Human, rat and mouse, WB, IP, IF, IHC and Elisa (https://www.ptglab.com/products/ANO2-Antibody-20647-1-AP.htm). Relevant citations:<br><br>(1)Guarascio DM, Gonzalez-Velandia KY, Hernandez-Clavijo A, Menini A, Pifferi S. Functional expression of TMEM16A in taste bud cells. J Physiol. 2021 Aug;599(15):3697-3714. doi: 10.1113/JP281645. Epub 2021 Jun 28. PMID: 34089532; PMCID: PMC8361675.<br>(2)Agostinelli E, Gonzalez-Velandia KY, Hernandez-Clavijo A, Kumar Maurya D, Xerxa E, Lewin GR, Dibattista M, Menini A, Pifferi S. A Role for STOML3 in Olfactory Sensory Transduction. eNeuro. 2021 Mar 12;8(2):ENEURO.0565-20.2021. doi: 10.1523/ENEURO.0565-20.2021. PMID: 33637538; PMCID: PMC7986538.<br><br>Anti-TMEM16B (1 citation), Human, rat and mouse, WB, IFC, LCI and IF (https://www.alomone.com/p/anti-anoctamin-6-extracellular/ACL-016). Relevant citations:<br><br>(1)Banerjee J, Leung CT, Li A, Peterson-Yantorno K, Ouyang H, Stamer WD, Civan MM. Regulatory Roles of Anoctamin-6 in Human Trabecular Meshwork Cells. Invest Ophthalmol Vis Sci. 2017 Jan 1;58(1):492-501. doi: 10.1167/iovs.16-20188. PMID: 28125837; PMCID: PMC5283088.

Eukaryotic cell lines

Policy information about cell lines and Sex and Gender in Research

Cell line source(s)	The cell line used in this study (HEK-293 and NG108-15) were purchased from European Collection of Authenticated Cell Cultures (ECACC) through its distributor Sigma Aldrich. NG180-15, Cat# 88112302; HEK-293, Cat# 85120602.
Authentication	All cell lines used in this study (HEK-293 and NG108-15) were authenticated by the supplier using standard authentication methos, such as short tandem repeat (STR) profiling. Additional information on the authentication procedures can be found on the supplier's website or product datasheets.
Mycoplasma contamination	The cell lines were not tested for mycoplasma
Commonly misidentified lines (See ICLAC register)	No commonly misidentified cell lines were used in this study

Plants

Seed stocks	NA
Novel plant genotypes	NA
Authentication	NA
